# Exploring Metabolic Obesity Phenotypes and Atherosclerotic Cardiovascular Disease Risk in Arab Adults

**DOI:** 10.1111/cob.70032

**Published:** 2025-06-27

**Authors:** Kaiser Wani, Balvir Kumar, Nasser M. Al‐Daghri, Shaun Sabico

**Affiliations:** ^1^ Chair for Biomarkers of Chronic Diseases, Biochemistry Department College of Science, King Saud University Riyadh Saudi Arabia; ^2^ Department of Biotechnology University Institute of Biotechnology, Chandigarh University Mohali India

**Keywords:** ASCVD, atherosclerotic cardiovascular disease, body mass index, cardiovascular risk assessment, metabolic syndrome, visceral adiposity index

## Abstract

This study investigates the 10‐year atherosclerotic cardiovascular disease (ASCVD) risk in different metabolic obesity phenotypes in Saudi adults. A cohort of 5460 adults (aged 40–79) was categorised based on metabolic status, body mass index (BMI), and visceral adiposity index (VAI). Using the ASCVD Risk Estimator Plus, the 10‐year risk scores were calculated and explored in different metabolic phenotypes. Females showed higher obesity prevalence, while males had a higher metabolically unhealthy phenotype prevalence. Despite being considered healthy by traditional metrics, individuals with Metabolically Healthy Obesity (MHO) exhibited significantly higher ASCVD risk scores compared to Metabolically Healthy Normal Weight (MHNW) counterparts (2.44 vs. 1.34 in females, *p* < 0.001; 9.60 vs. 6.72 in males, *p* = 0.008). When obesity was defined by BMI, in men, MHO showed a substantially higher age‐adjusted odds ratio (OR) for greater ASCVD risk than MHNW (OR = 2.04, 95% CI 1.3–3.3, *p* = 0.003). However, when obesity was characterised by VAI rather than BMI, ASCVD risk in metabolically healthy with high VAI (MHHV), equivalent to MHO, was similar to its normal VAI counterpart, independent of gender (OR = 0.92, 95% CI 0.7–1.2, *p* = 0.55 for females; OR = 1.23, 95% CI 0.9–1.7, *p* = 0.25 for men). The study provides insights into ASCVD risk in multiple metabolic and obesity phenotypes among Saudi individuals, indicating that VAI outperforms BMI in identifying the metabolically healthy obese phenotype.

**Summary**

- What is already known about this subject
  ○ ASCVD risk scores for metabolic obesity phenotypes in earlier studies showed disparity in results, as the obesity index used in most of these studies is the traditional index of BMI.
- What this study adds
  ○ Apart from the traditional obesity index (BMI) used a novel index of visceral adiposity index (VAI)
  ○ Proposed the use of VAI to define the ‘Metabolically Healthy Obesity’ phenotype to help explain the ‘Obesity Paradox’ proposed in the literature

## 1 Introduction

Globally, ischemic heart disease (IHD) and stroke remain the primary causes of morbidity and mortality as cardiovascular diseases (CVDs) [1, 2]. In 2019, an alarming 18.6 million fatalities were linked to CVD, underscoring its widespread impact in a global analysis [3]. IHD and stroke are the second and fourth leading causes of death in Saudi Arabia, respectively, and they place a heavy financial strain on the healthcare system [4]. Addressing the challenge, preventive cardiology prioritises individuals at high risk of CVD by an assessment of a risk score, such as the one done in atherosclerotic cardiovascular disease (ASCVD) Risk Estimator Plus, recommended by the American College of Cardiology and the American Heart Association [5]. This tool calculates the 10‐year probability of an ASCVD event in people without a history of CVD [6, 7], and is based on traditional risk factors such as obesity, hypertension, diabetes, dyslipidemia, smoking, etc. [8, 9].

Obesity and metabolic syndrome profoundly influence CVD, increasing risk and leading to unfavourable outcomes [10]. Obesity, characterised by excess body fat, is recognised as an independent risk factor for CVD [11, 12]. It has a role in the development of diseases, including insulin resistance, dyslipidemia, and hypertension, which together make up the metabolic syndrome cluster [13, 14]. A thorough understanding of the intricate interactions between metabolic health, obesity, and the risk of ASCVD is thus necessary to effectively address the cardiovascular consequences of obesity and metabolic syndrome. This understanding must be put into practice through active engagement, in‐depth education, and collaborations [15, 16]. Historical evidence suggests the substantial benefits of adopting such an approach, demonstrating a noteworthy reduction in the risk of both cardiovascular and microvascular events [17].

The intricate relationship between obesity and cardiovascular risk introduces a concept called the obesity paradox, which has received much attention lately, wherein individuals with obesity, defined by body mass index (BMI), with CVD sometimes exhibit better long‐term prognoses than their lean counterparts [18, 19]. Similarly, certain individuals with obesity, also referred to as metabolically healthy obese (MHO), do not exhibit cardiometabolic abnormalities inherent to obesity, while the ones with these abnormalities are called metabolically unhealthy obese (MUO) [20, 21, 22]. There is, however, a debate as to whether individuals with MHO are truly ‘protected’ from metabolic abnormalities or whether this form of obesity is a transient phase of MUO [23, 24, 25]. Traditionally, these phenotypes of obesity have been defined using BMI as a surrogate marker of obesity, and over the years, reports have highlighted the limitations of using this marker to describe the distribution of body fat [26, 27]. A non‐traditional novel marker of visceral adiposity and adipose tissue function, called VAI, according to some reports, yielded better results in predicting elevated ASCVD risk [28].

Taken together the above concepts, it is interesting to study the ASCVD risk in phenotypes of metabolic status and obesity, defined either by the traditional or the novel adiposity indices. Limited studies have, however, tried to explore this nuanced relationship, especially in this population. The present study thus delves into this intricate relationship between obesity phenotypes and their ASCVD risk scores by building upon a robust cohort of 5460 Saudi adults in the age‐range of 40–79 years. The hypothesis was that these phenotypes, defined by combinations of metabolic status, BMI, and VAI, may shed light on the intricate landscape of cardiovascular risk in a diverse population.

## 2 Materials and Methods

### 2.1 Study Design and Participants

**FIGURE 1:**
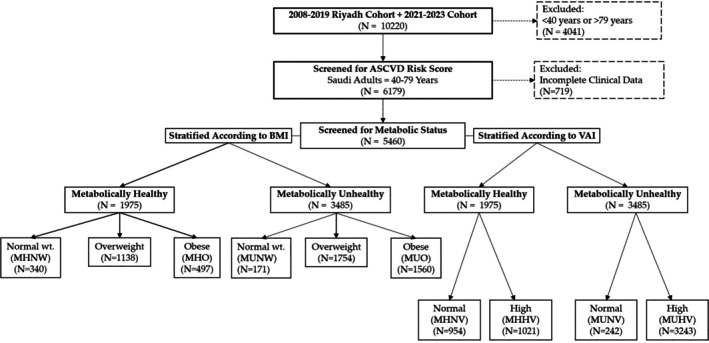
Flow chart of the study participants.

A total of 10 220 Saudi adults (63.5% females) were initially screened for this observational study from the database of the Center for Biomarkers of Chronic Diseases (CBCD) biobank, collected from the year 2008 as ‘RIYADH Cohort’. In brief, this cohort encompasses comprehensive demographic and clinical data obtained from Saudi residents within the city of Riyadh and was primarily facilitated through collaboration with the Ministry of Health, Riyadh, SA. The recruitment process primarily involved primary healthcare centres and schools to assemble a diverse dataset for epidemiological investigations [29, 30]. From this 10 220, in the first screening, a total of 4041 subjects (62.7% females) with ages < 40 and > 79 years were excluded as the ASCVD risk score estimation using the ACC/AHA calculator required an age range of 40–79 years. From the remaining 6179 subjects, the data from a total of 719 subjects was excluded because of a lack of information in one or more of the parameters required to either calculate the ASCVD risk score or to categorise the subjects into metabolically healthy or unhealthy phenotypes. The data from the remaining 5460 subjects (age range 40–79 years, 64.5% females) were used to explore the objectives of this study. The Institutional Review Board (IRB) at the College of Medicine, KSU (E‐22‐7142), approved the study. A comprehensive flow chart detailing the selection and exclusion from the total database of 10 220 subjects is illustrated in Figure 1.

### 2.2 Socio‐Demographic, Clinical, and Biochemical Evaluations

A comprehensive assessment of the sociodemographic and clinical information of the study participants has been followed as a routine for any epidemiological study conducted by the CBCD. In brief, a standard questionnaire was administered to each participant and collected information about demographics like marital status, income group, family history, individual medical history, current/past smoking history, etc. Clinical information like age at recruitment, gender, weight (in kilograms), height (in centimetres), waist circumference, and hip circumference (in centimetres) was collected using standard procedures. The blood pressure information was collected by the recruiting personnel using digital portable blood pressure monitors as an average of two readings recorded over 15‐min durations.

At each recruitment phase, the recruiting personnel collected a minimum of 10‐hr fasting blood samples from each participant, and these were transported immediately to the CBCD lab where they were processed for serum, aliquoted, and stored at optimum conditions before analysis. A standard biochemistry analyser (Konelab 20XT, Thermo Scientific, Vantaa, Finland) was utilised to assess circulating concentrations of glucose, total cholesterol, HDL cholesterol, and triglycerides. This involved the use of commercially available bioassay kits (reference numbers 981 379, 981 812, 981 823, and 981 301, respectively). These estimations were utilised for defining the metabolic health of each participant. Also, circulating insulin, utilised for estimating insulin resistance HOMA‐IR, was estimated using ELISA.

### 2.3 Calculations and Definitions of Phenotypes

LDL‐cholesterol was derived using the Friedewald equation [31] as below:

LDL‐Cholesterol (mmol/L) = Total cholesterol (mmol/L) − HDL‐cholesterol (mmol/L) − triglycerides (mmol/L)/2.2.

HOMA‐IR [32], as a measure of insulin resistance, was derived from fasting glucose and insulin concentrations as below:

HOMA‐IR = (insulin (μU/L) × fasting glucose (nmol/L))/22.5.

BMI was calculated as the ratio of weight to the square of height in meters, while WHR was determined as the ratio of waist to hip circumference in centimetres. The VAI [33], as an indicator of visceral adiposity, was derived using the following formula:

Males: ((waist/39.68 + (1.88 × BMI)) × (triglyceride/1.03) × (1.31/HDL‐C) and females: ((waist/36.58 + (1.89 × BMI)) × (triglyceride/0.81) × (1.52/HDL).

The optimal VAI cut‐offs, used in this study, were 1.83 in males and 1.58 in females [34].

The criteria for evaluating the status of metabolic unhealthy were derived using the NCEP ATPIII definitions for MHO [35], as the presence of at least three risk factors below:

- Abdominal obesity, defined as WC ≥ 88 cm in females and WC ≥ 102 cm in males
- Elevated FBG ≥ 5.6 mmol/L or taking diabetes medications or diagnosis of diabetes
- Elevated blood pressure of ≥ 130/85 mmHg or taking blood pressure medications
- Low HDL‐cholesterol of < 1.29 mmol/L in females and < 1.03 mmol/L in males
- Elevated TG levels of ≥ 1.7 mmol/L
  Metabolic status + BMI or metabolic status + VAI were used to define the phenotypes of the study subjects (Figure 1).

### 2.4 Predicted 10‐Year ASCVD Risk Score and Categories

The ASCVD Risk Estimator Plus, developed by the American College of Cardiology and the American Heart Association (ACC/AHA), stands as an online tool designed to assess the potential occurrence of ASCVD events over the next decade in individuals without existing CVD. This innovative tool relies on a comprehensive set of well‐established risk factors to make estimations. The incorporated risk factors include age, gender, systolic and diastolic blood pressures, circulating levels of total cholesterol, HDL cholesterol, and LDL cholesterol, as well as considerations of race, diabetes history, smoking status, and the use of statins, hypertension treatment, or aspirin [5]. To conduct the analysis, participant data collected at the recruitment were systematically input into the ASCVD Risk Estimator Plus. Subsequently, the tool generated a current 10‐year ASCVD risk score for each participant. The resulting scores were then categorised into distinct risk levels: low risk (< 7.5%), intermediate risk (7.5%–19.9%), and high risk (≥ 20%).

### 2.5 Statistical Analysis

Continuous variables were presented as mean ± standard deviation (SD) and median (quartile 1, quartile 3) for normal and non‐normal variables (like VAI, 10‐year ASCVD risk score, triglycerides, LDL‐cholesterol, insulin, HOMA‐IR), respectively, while categorical variables were expressed as frequencies (percentages). Gender‐specific comparisons were conducted using t‐tests for continuous variables and Chi‐square tests for categorical variables. Independent *t*‐test, Mann–Whitney *U* test and Chi‐square tests, respectively, for normal, non‐normal, and categorical variables, were used for intergroup comparisons of obesity phenotypes. The non‐normal variables, including VAI and 10‐year ASCVD risk scores, were log‐transformed before further analysis. A bivariate correlation analysis was done to check associations of the 10‐year ASCVD risk score with other measured variables. The subjects were divided into phenotypes based on the cut‐offs for BMI, VAI, and metabolic status, and logistic regression analysis was employed to calculate odds ratios (ORs), generating sex‐specific ORs with 95% confidence intervals of having higher ASCVD risk vs. low ASCVD risk in these phenotypes. OR^a^ represented the age‐adjusted model. All analyses were conducted using SPSS 21.0 for Windows (SPSS Inc., Chicago, IL, USA), with statistical significance set at *p* < 0.05. Microsoft Excel 2016 was used to plot the graphs.

## 3 Results

### 3.1 Characteristics of the Study Participants

The general characteristics of the study subjects are shown in Table 1. Mean age and BMI were 52.96 ± 9.3 years and 29.95 kg/m^2^ ± 4.7, respectively. Of the total subjects, 3519 (64.5%) were females and 1941 (35.5%) were males. The proportion of subjects classified as overweight or obese was 87.9% in females and 79.8% in males (*p* < 0.001). They had a comparable but high proportion of ones with high VAI scores (78.4% vs. 77.5%, *p* = 0.46). Females had a higher proportion of those with obesity by BMI and VAI than males (42.7% vs. 25.6%), while males had a high proportion of individuals with high VAI but normal BMI (13.7% vs. 8.5%). Males presented a higher proportion of ones with a metabolically unhealthy phenotype compared to females (67.1% vs. 62%, *p* < 0.001). Males had a higher proportion of married and more income groups than females: almost 40% and 25% of the total subjects suffered from diabetes and elevated BP, respectively. When divided into ASCVD risk score categories, males had a much higher proportion of ones with intermediate to high 10‐year ASCVD risk compared to females (70.3% vs. 21.6%, *p* < 0.001).

**TABLE 1:** Characteristics of study subjects.

	All (5460)	Females (3519)	Males (1941)	*p*
Age	52.96 ± 9.3	52.23 ± 8.8	54.28 ± 9.9	< 0.001
Gender				
Female	3519 (64.5)	3519 (100)	—	—
Male	1941 (35.5)	—	1941 (100)	
BMI				
BMI (kg/m^2^)	29.95 ± 4.7	30.70 ± 4.8	28.60 ± 4.2	< 0.001
Normal	820 (15)	426 (12.1)	394 (20.2)	< 0.001
Overweight	2149 (39.4)	1224 (34.8)	925 (47.7)	
Obese	2491 (45.6)	1869 (53.1)	622 (32.1)	
VAI				
VAI (score)	2.74 (1.8, 4.5)	2.59 (1.7, 4.2)	3.07 (1.9, 5.2)	< 0.001
Normal	1196 (21.9)	760 (21.6)	436 (22.5)	0.46
High	4264 (78.1)	2759 (78.4)	1505 (77.5)	
Metabolic health status				
Metabolically healthy	1975 (36.2)	1336 (38.0)	639 (32.9)	< 0.001
Metabolically unhealthy	3485 (63.8)	2183 (62.0)	1302 (67.1)	
Smoking status				
	665 (12.2)	196 (5.6)	469 (24.2)	< 0.001
Demographics^a^				
Marital status				
Single	187 (5.4)	124 (5.7)	63 (5.1)	< 0.001
Married	2719 (79.2)	1568 (71.8)	1151 (92.2)	
Divorced	251 (7.3)	238 (10.9)	13 (1)	
Widowed	276 (8)	254 (11.6)	22 (1.8)	
Income				
No income	223 (14.1)	208 (35.5)	15 (1.5)	< 0.001
< 5 K	398 (25.2)	103 (17.6)	295 (29.7)	
5–10 K	530 (33.6)	228 (38.9)	302 (30.4)	
10–20 K	182 (11.5)	19 (3.2)	163 (16.4)	
> 20 K	246 (15.6)	28 (4.8)	218 (22)	
Family history^a^				
Diabetes	1831 (62.2)	1136 (63.9)	695 (59.7)	0.02
Hypertension	1302 (45.7)	836 (48.9)	466 (40.8)	< 0.001
Dyslipidemia	209 (7.9)	158 (10.3)	51 (4.6)	< 0.001
Asthma	304 (11.7)	186 (12.5)	118 (10.6)	0.15
CVD	177 (6.7)	128 (8.4)	49 (4.4)	< 0.001
Cancer	35 (1.3)	21 (1.4)	14 (1.3)	0.72
Health status				
Diabetes	2102 (39.1)	1198 (34.7)	904 (47)	< 0.001
Elevated BP	387 (24.9)	199 (20.9)	188 (31.2)	< 0.001
Dyslipidemia	117 (7.5)	43 (4.5)	74 (12.3)	< 0.001
Asthma	120 (7.7)	65 (6.8)	55 (9.1)	0.10
Arthritis	150 (9.7)	142 (14.9)	8 (1.3)	< 0.001
MetS	3485 (63.8)	2183 (62)	1302 (67.1)	< 0.001
ASCVD				
10‐year ASCVD score	4.99 (2.1, 13.0)	3.14 (1.5, 6.5)	13.99 (6.2, 25.7)	< 0.001
ASCVD risk status				
Low risk	6476 (67.2)	2756 (78.3)	578 (29.7)	< 0.001
Intermediate	2277 (23.6)	603 (17.1)	683 (35.2)	
High	878 (9.1)	160 (4.5)	680 (35.1)	

*Note*: Data was presented as mean ± SD, median (Q1, Q3), and *N* (%) for Gaussian continuous, non‐Gaussian continuous, and categorical variables, respectively. The *p* value represents the difference between the sexes.

^a^ The data that was missing in these variables and the % was calculated from the total of ones with this data.

**FIGURE 2:**
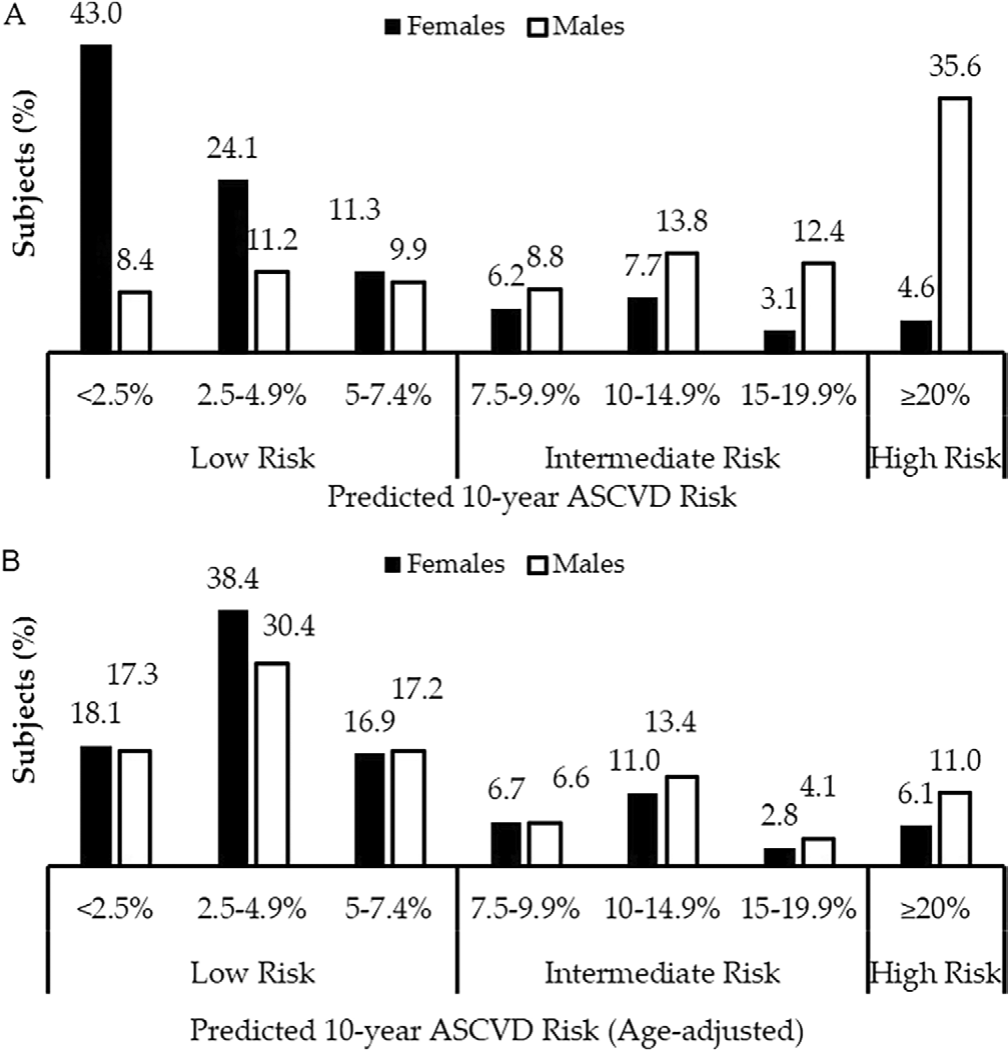
Prevalence of the Predicted 10‐year ASCVD risk in the study population.

The prevalence of the predicted 10‐year ASCVD risk score in the study population is shown in Figure 2.

### 3.2 Characteristics in Groups Divided According to Metabolic Status and BMI

The study subjects were divided based on metabolic status and BMI into four groups, and the data of anthropometric and biochemical indices in the groups were compared with the ones with normal metabolic status and normal BMI (MHNW) (Table 2). In females, the median VAI score in the MHO group was not significantly different from the MHNW group, while in males, lower median VAI scores were observed in the MHO group compared to the MHNW group (1.59 vs. 1.67, *p* = 0.71 in females, and 1.50 vs. 1.79, *p* = 0.04 in males). MUO group, on the other hand, in both sexes, had significantly higher median VAI scores compared to the MHNW group (3.26 vs. 1.67, *p* < 0.001 in females, and 3.68 vs. 1.79, *p* < 0.001 in males). No statistical difference for HOMA‐IR, a measure of insulin resistance, was observed between MHO and MHNW groups in both sexes (2.00 vs. 1.55, *p* = 0.117 in females, and 2.90 vs. 1.79, *p* = 0.73 in males) while between MUO and MHNW groups, MUO group in both sexes showed significantly higher HOMA‐IR values compared to the MHNW group (4.05 vs. 1.55, *p* < 0.001 in females, and 6.05 vs. 1.79, *p* < 0.001 in males). The 10‐year ASCVD risk scores were also observed between these groups and we found the ASCVD scores were significantly higher in both MHO and MUO groups when compared to the MHNW groups, irrespective of sex (in females, 2.44 vs. 1.34, *p* < 0.001 and 3.98 vs. 1.34, *p* < 0.001 when compared between MHO vs. MHNW, and MUO vs. MHNW respectively; and in males, 9.60 vs. 6.72, *p* = 0.008, and 15.40 vs. 6.72, *p* < 0.001 when compared to MHO vs. MHNW, and MUO vs. MHNW respectively).

**TABLE 2:** Characteristics of study subjects in different phenotypes of metabolic status and BMI.

Characteristics	Metabolic phenotypes				*p* values compared to MHNW		
	MHNW	MHO	MUNW	MUO	Vs. MHO	Vs. MUNW	Vs. MUO
Females							
*N*	254	511	172	1358			
Age (years)	51.34 ± 9.6	50.29 ± 7.7	55.35 ± 10.1	52.91 ± 8.6	0.10	< 0.001	0.009
BMI (kg/m^2^)	22.8 ± 1.6	33.78 ± 2.9	23.18 ± 1.4	34.69 ± 3	< 0.001	0.01	< 0.001
VAI	1.67 (1.1, 2.2)	1.59 (1.2, 2.2)	3.79 (2.8, 5.8)	3.26 (2.2, 5.1)	0.71	< 0.001	< 0.001
Waist (cm)	80.08 ± 11.6	96.47 ± 13.3	90.56 ± 13	104.02 ± 11.3	< 0.001	< 0.001	< 0.001
Hips (cm)	92.85 ± 12.5	109.58 ± 12.7	96.53 ± 13.9	111.91 ± 12.2	< 0.001	0.005	< 0.001
WHR	0.87 ± 0.1	0.89 ± 0.1	0.95 ± 0.1	0.94 ± 0.2	0.06	< 0.001	< 0.001
Systolic BP (mmHg)	115.24 ± 14.9	118.04 ± 12.8	127.56 ± 17.5	129.73 ± 16.2	0.006	< 0.001	< 0.001
Diastolic BP (mmHg)	73.16 ± 9.1	74.73 ± 8.1	78.26 ± 9.3	80.01 ± 10.5	0.01	< 0.001	< 0.001
T. cholesterol (mmol/L)	5.38 ± 1.3	5.21 ± 1	5.33 ± 1.3	5.23 ± 1.2	0.04	0.69	0.06
Glucose (mmol/L)	6.02 ± 3.2	5.73 ± 2.8	8.96 ± 4.2	8.45 ± 4.1	0.19	< 0.001	< 0.001
HDL‐cholesterol (mmol/L)	1.35 ± 0.5	1.3 ± 0.4	0.98 ± 0.4	0.99 ± 0.4	0.12	< 0.001	< 0.001
Triglycerides (mmol/L)	1.14 (0.9, 1.4)	1.21 (1, 1.5)	1.84 (1.5, 2.4)	1.81 (1.3, 2.3)	0.13	< 0.001	< 0.001
LDL‐cholesterol (mmol/L)	3.47 (2.7, 4.1)	3.28 (2.7, 3.9)	3.36 (2.5, 4.1)	3.27 (2.6, 4)	0.42	0.63	0.16
Insulin (μU/mL)	6.27 (3.2, 13)	8.56 (4.7, 13.8)	8.53 (5.5, 13.7)	11.93 (7.1, 18.9)	0.15	0.13	< 0.001
HOMA‐IR	1.55 (0.7, 3.1)	2.00 (1.3, 3.3)	2.69 (1.6, 5.8)	4.05 (2.3, 7.1)	0.12	0.003	< 0.001
ASCVD score	1.34 (0.8, 3.2)	2.44 (1.5, 4.3)	3.91 (1.6, 8.1)	3.98 (2.1, 7.9)	< 0.001	< 0.001	< 0.001
Males							
*N*	206	111	188	511			
Age (years)	53.85 ± 10.8	49.5 ± 8.3	57.84 ± 10	53.99 ± 9.4	< 0.001	< 0.001	0.87
BMI (Kg/m^2^)	22.9 ± 1.7	32.94 ± 2.7	23.57 ± 1.3	33.7 ± 2.7	< 0.001	< 0.001	< 0.001
VAI	1.79 (1.3, 2.7)	1.50 (1.1, 2.2)	3.77 (2.6, 6.2)	3.68 (2.4, 5.9)	0.04	< 0.001	< 0.001
Waist (cm)	85.42 ± 10.9	103.84 ± 12.2	92.28 ± 13.9	110.18 ± 12	< 0.001	< 0.001	< 0.001
Hips (cm)	92.36 ± 11.4	108.95 ± 14.7	94.84 ± 13.9	111.72 ± 12.2	< 0.001	0.05	< 0.001
WHR	120.78 ± 14.1	121.95 ± 11.5	131.15 ± 15	132.97 ± 14.7	0.43	< 0.001	< 0.001
Systolic BP	76.14 ± 7.8	77.22 ± 10.1	80.37 ± 9	82.32 ± 9.2	0.27	< 0.001	< 0.001
Diastolic BP	0.93 ± 0.1	0.97 ± 0.2	0.98 ± 0.1	0.99 ± 0.1	0.008	< 0.001	< 0.001
T. cholesterol (mmol/L)	5.08 ± 1.1	5.26 ± 1.3	5.25 ± 1.5	5.28 ± 1.4	0.17	0.18	0.06
Glucose (mmol/L)	6.76 ± 3.8	6.2 ± 3.3	10 ± 4.7	9.07 ± 4.3	0.17	< 0.001	< 0.001
HDL‐cholesterol (mmol/L)	0.96 ± 0.4	1.07 ± 0.3	0.77 ± 0.3	0.79 ± 0.3	0.004	< 0.001	< 0.001
Triglycerides (mmol/L)	1.27 (1, 1.6)	1.24 (1, 1.5)	2.02 (1.5, 2.8)	2.12 (1.6, 2.9)	0.33	< 0.001	< 0.001
LDL‐cholesterol (mmol/L)	3.36 (2.7, 4.2)	3.45 (2.8, 4.2)	3.3 (2.5, 4.2)	3.35 (2.6, 4.1)	0.44	0.12	0.18
Insulin (μU/mL)	7.53 (4.1, 17.8)	9.19 (3.9, 15.2)	8.81 (6, 16.4)	14.81 (8.3, 26.6)	0.93	0.26	< 0.001
HOMA‐IR	1.79 (0.9, 5.7)	2.90 (1.3, 5)	3.66 (2.2, 9.1)	6.05 (3, 10.5)	0.73	0.002	< 0.001
ASCVD score	6.72 (2.9, 17.4)	9.6 (5.3, 18.7)	16.08 (8.1, 28.7)	15.4 (8.2, 27.1)	0.008	< 0.001	< 0.001

*Note*: Data were presented as mean ± SD, and median (Q1, Q3) for Gaussian continuous and non‐Gaussian continuous variables, respectively. *p* value represents the differences compared to the control (MHNW group). *p* < 0.05 was considered significant.

**FIGURE 3:**
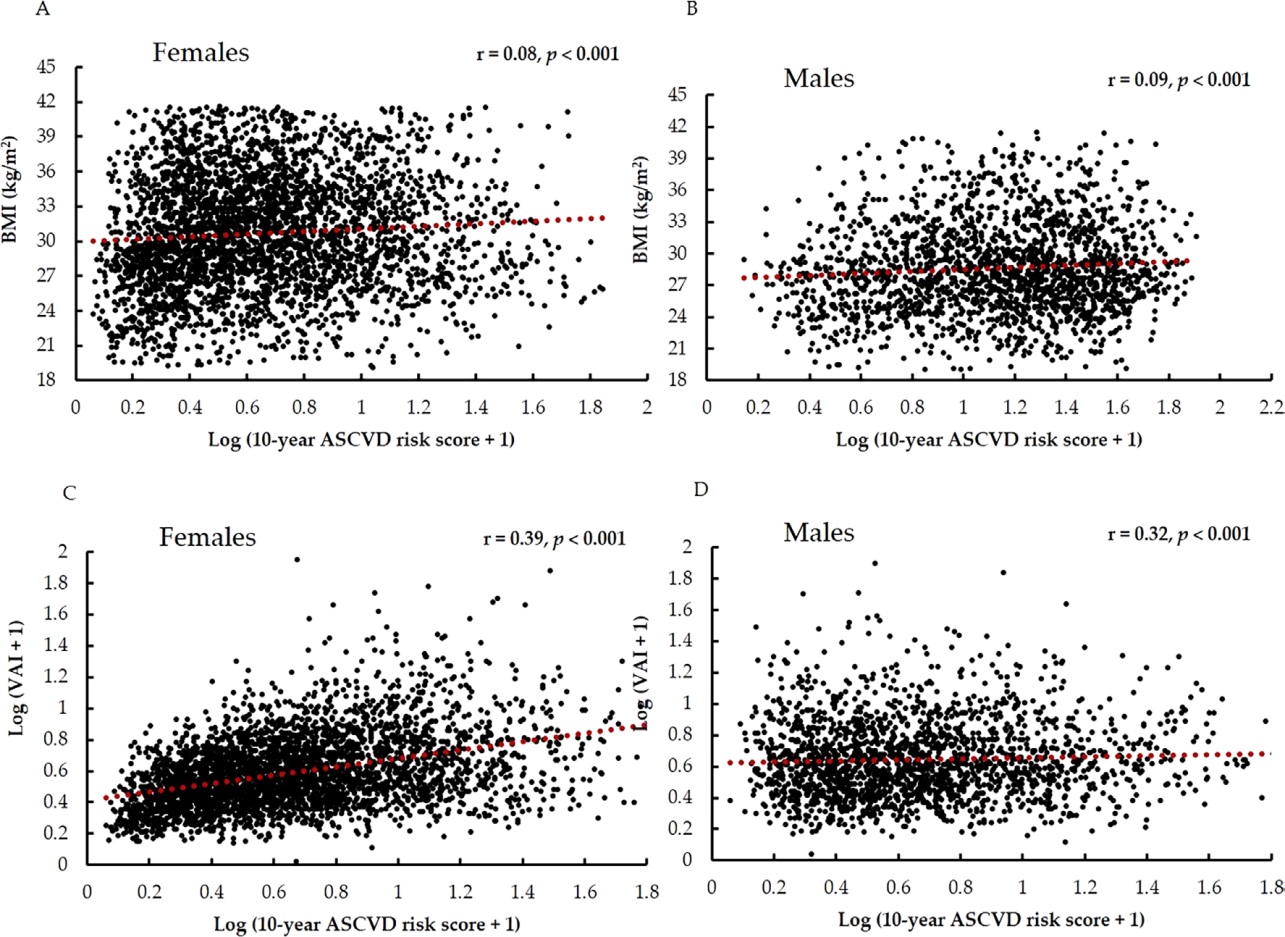
Scatterplots depicting bivariate correlations for 10‐year ASCVD risk scores with BMI (A‐females and B‐males) and VAI (C‐females and D‐males).

A bivariate correlation analysis of the ASCVD risk score with other parameters was done and presented as Table S1. The bivariate correlation of ASCVD risk scores with BMI and VAI was presented as scatterplots in Figure 3.

### 3.3 Association of ASCVD Risk With Different Phenotypes

ASCVD risk scores were divided into ones with low (< 7.5%) and intermediate to high risk (≥ 7.5%). A logistic regression with ASCVD risk category as the dependent variable and different phenotypes as independent variables was done to get the age‐adjusted odds of having intermediate to high risk ASCVD compared to low risk, using healthy phenotype as reference. The data was presented as Odds Ratio (OR) and its 95% confidence intervals (95% CI) (Table 3). The age‐adjusted odds of having intermediate to high‐risk 10‐year ASCVD score compared to low‐risk in females and males were significantly higher than with metabolically unhealthy phenotypes (OR = 1.58, 95% CI of 1.3–1.8, *p* < 0.001 for females and OR = 1.90, 95% CI of 1.6–2.3, *p* < 0.001 for males). According to VAI status, subjects with high VAI showed significantly higher odds of having higher ASCVD risk than those with normal VAI (OR = 1.21, 95% CI of 1.0–1.5, *p* = 0.02 for females and OR = 1.49, 95% CI of 1.2–1.9, *p* < 0.001 for males).

**TABLE 3:** 10‐year ASCVD risk scores and age‐adjusted odds ratios among different phenotypes according to sex.

Phenotypes	FEMALES				MALES			
	10‐year ASCVD risk score		Logistic regression		10‐year ASCVD risk score		Logistic regression	
	Low risk	Intermediate to high risk	Age‐adjusted OR (95% CI)	*p*	Low risk	Intermediate to high risk	Age‐adjusted OR (95% CI)	*p*
Metabolic health status								
Healthy	1206 (90.3)	130 (9.7)	1.0		321 (55.5)	318 (23.3)	1.0	
Unhealthy	1550 (71.0)	633 (29.0)	1.58 (1.3, 1.8)	< 0.001	257 (44.5)	1045 (76.7)	1.90 (1.6, 2.3)	< 0.001
BMI								
Normal	358 (84.0)	68 (16.0)	1.0		153 (26.5)	241 (17.7)	1.0	
Overweight	932 (76.1)	292 (23.9)	1.05 (0.8, 1.3)	0.68	266 (46.0)	659 (48.3)	0.80 (0.6, 1.0)	0.06
Obese	1466 (78.4)	403 (21.6)	0.87 (0.7, 1.1)	0.25	159 (27.5)	463 (34.0)	0.61 (0.5, 0.8)	< 0.001
VAI								
Normal	688 (90.5)	72 (9.5)	1.0		227 (52.1)	209 (47.9)	1.0	
High	2068 (75.0)	691 (25.0)	1.25 (1.0, 1.5)	0.02	351 (23.3)	1154 (76.7)	1.49 (1.2, 1.9)	< 0.001
Metabolic health according to BMI status								
MHNW	232 (91.3)	22 (8.7)	1.0		108 (52.4)	98 (47.6)	1.0	
MHO	458 (89.6)	53 (10.4)	0.79 (0.5, 1.1)	0.20	39 (35.1)	72 (64.9)	1.42 (1.2, 1.7)	0.01
MUNW	126 (73.3)	46 (26.7)	2.06 (1.4, 3.1)	< 0.001	45 (23.9)	143 (76.1)	2.03 (1.4, 3.0)	< 0.001
MUO	1008 (74.2)	350 (25.8)	1.38 (1.0, 1.9)	0.04	120 (23.5)	391 (76.5)	1.98 (1.7, 2.4)	< 0.001
Metabolic health according to VAI status								
MHNV	578 (93.2)	42 (6.8)	1.0		194 (58.1)	140 (41.9)	1.0	
MHHV	628 (87.7)	88 (12.3)	0.92 (0.7, 1.2)	0.55	127 (41.6)	178 (58.4)	1.23 (0.9, 1.7)	0.25
MUNV	110 (78.6)	30 (21.4)	2.30 (1.6, 3.4)	< 0.001	33 (32.4)	69 (67.6)	2.42 (1.5, 3.8)	< 0.001
MUHV	1440 (70.5)	603 (29.5)	1.71 (1.4, 2.1)	< 0.001	224 (18.7)	976 (81.3)	2.08 (1.6, 3.8)	< 0.001

*Note*: The data was presented as *N* (%) for low risk and intermediate to high risk for individual phenotypes. Logistic regression analysis was done for odds of having intermediate to high risk ASCVD compared to low risk ASCVD in different phenotypes taking healthy phenotype as reference and the data was presented as age‐adjusted Odds Ratio (OR) and its 95% confidence intervals (95% CI). *p* < 0.05 was taken as significant.

For the phenotypes defined by BMI, we found significantly higher age‐adjusted odds of higher ASCVD risk scores in MUO vs. MHNW phenotypes in all subjects (OR = 1.38, 95% CI of 1.0–1.9, *p* = 0.04 for females and OR = 1.98, 95% CI of 1.7–2.4, *p* < 0.001 for males). MHO phenotype in females showed comparable odds of having higher vs. lower ASCVD risk (age‐adjusted OR = 0.79, 95% CI of 0.5–1.1, *p* = 0.20). However, in males, significantly higher odds were seen for MHO vs. MHNW (age‐adjusted OR = 1.42, 95% CI of 1.2–1.7, *p* = 0.01).

For phenotypes defined by metabolic health status + VAI, significantly higher age‐adjusted odds of having higher ASCVD risk score were observed in those with metabolically unhealthy with high VAI (MUHV) phenotype compared to MHNV, irrespective of gender (OR = 1.7, 95% CI of 1.4–2.1, *p* < 0.001 for females and OR = 2.1, 95% CI of 1.6–3.8, *p* < 0.001 for males). However, between MHHV (corresponding to MHO) and MHNV phenotypes, the age‐adjusted ORs were insignificant.

## 4 Discussion

The study assessed the cardiovascular risk scores in different phenotypes defined by metabolic status and obesity, and we used the traditional index of BMI as well as the novel index of VAI to categorise obesity. The objective was to investigate primarily the 10‐year ASCVD risk, through a risk assessment calculator proposed by ACC/AHA, in MHO and MUO phenotypes of obesity when compared with the MHNW phenotype. The study was designed since, to the best of our knowledge, no studies have been reported in this population where ASCVD risk was investigated in obesity phenotypes and secondly, because there have been controversies concerning the existence of MHO or ‘fat but fit’ phenotype. The findings revealed a sex‐specific relationship between 10‐year ASCVD risk and obesity phenotypes, with males exhibiting significantly higher age‐adjusted odds of elevated ASCVD risk compared to MHNW counterparts when obesity was defined by BMI. However, between these phenotypes, we found insignificant odds of higher vs. lower ASCDV risk in both genders when obesity was defined by VAI, indicating that VAI may be a better index of adiposity for defining MHO.

Our data showed a significant prevalence of overweight and obesity among individuals, with noteworthy disparities between sexes in these adiposity patterns. One of the probable reasons may be the composition of the study population with adults in the age range of 40–79 years, as ASCVD risk assessment through the ACC/AHA calculator requires this age range. Besides, the study population had a greater prevalence of obesity in females (64.5%), as measured by both BMI and the VAI. The observed greater prevalence of obesity in females is consistent with more general trends reported in the literature, where women often display higher rates of obesity than men across different demographics [36]. The complex relationship of biological, hormonal, and socio‐cultural factors [37] contributes to gender differences in adiposity patterns. The gender‐specific variation in adiposity also signifies the critical importance of considering sex‐specific factors in cardiovascular risk assessment [38].

The higher prevalence of metabolically unhealthy phenotype and higher overall ASCVD risk scores in males versus females, reported in this study, despite the higher prevalence of obesity in females, adds another layer of complexity to our findings. Though obesity is a major independent risk factor for metabolic syndrome and CVD, a phenomenon known as ‘obesity paradox’ has been reported in the recent past, where individuals with overweight or obesity may have better CVD outcomes or survival rates compared to their lean counterparts [39, 40, 41]. Some theories to explain the reason include cardio‐protective effects of increased body mass due to increased nutritional reserves [42], and improved insulin sensitivity for mitigating negative impacts of obesity [43] have been proposed. Our results of the highest age‐adjusted odds of elevated ASCVD risk in metabolically unhealthy but non‐obese phenotypes (as MHNW), irrespective of gender, are in line with similar findings [44, 45] and support the obesity paradox theory of potential protective mechanisms in individuals with obesity, but raise questions whether obesity defined by BMI itself justifies explanation into the metabolic status and risk for CVD outcomes [46]. While the literature has extensively explored the adverse cardiovascular effects of obesity, the phenomenon of metabolic health in the context of obesity, defined by BMI, has garnered increasing attention and warrants further exploration.

The multifaceted relationship between HOMA‐IR as a marker of insulin resistance and different metabolic and obesity phenotypes, observed in this study, highlights the complexity of these interactions. The lack of significant differences in HOMA‐IR between MHO and MHNW groups suggests that insulin resistance may not be a distinguishing factor in these phenotypes. The absence of marked differences in insulin resistance between individuals with MHO and MHNW challenges the conventional notion that obesity, even in a metabolically healthy state, is universally associated with insulin resistance [47]. These observations regarding HOMA‐IR, however, align with studies proposing that not all individuals with obesity exhibit insulin resistance, emphasising the heterogeneity within the obese population concerning metabolic health [48]. In contrast, the MUO phenotype had substantially higher HOMA‐IR values, irrespective of gender, supporting the idea that metabolic disturbances in the context of obesity lead to decreased insulin sensitivity. The processes of dysfunctional adipose tissue, release of pro‐inflammatory cytokines, and elevated free fatty acids may play leading roles in the exacerbation of insulin resistance in these phenotypes [49].

In the present study, some interesting sex‐dimorphic trends were observed, especially in the MHO group, which is often seen as paradoxical since it is associated with reported metabolic health while being obese. Interestingly, the MHO phenotype in males showed significantly higher odds of elevated ASCVD risk when compared to individuals with MHNW. These findings, at least in males, are in line with multiple reports which challenge the notion that individuals with obesity, especially those classified as MHO, might be protected from adverse cardiovascular outcomes [50, 51]. Several definitions has been used to define MHO in other similar reports and also ones where the cardiovascular risk of this phenotype was insignificant compared to the MHNW phenotype [52, 53, 54], however, most of these definitions vary in how metabolic status was defined without exploring much into how the definition of obesity significantly impacts this relationship. The observations in this study highlighted the need for a nuanced approach that recognises the heterogeneity within the population with obesity, considering variations in metabolic health status [55, 56].

Gender‐specific variations that shed light on the complex relationships among metabolic health, obesity defined by BMI, and visceral adiposity were also revealed by this study. For phenotypes defined by metabolic status and obesity by VAI, logistic regression analysis revealed that the age‐adjusted odds of higher vs. lower ASCVD risk were comparable between MHHV and the MHNV groups, irrespective of gender, aligning with the definition of metabolically healthy obese phenotype. The findings suggest that the utilisation of VAI as a measure of obesity, for defining MHO, provides a more comprehensive assessment of adiposity, capturing not only overall body composition but also accounting for visceral fat distribution [57]. VAI, a novel marker of visceral adiposity and adipose tissue function, has demonstrated its efficacy in predicting cardiovascular risk beyond traditional measures like BMI [58]. Our findings also confirmed this as phenotypes with high VAI significantly increased the odds of elevated ASCVD risk, beyond metabolic status, in both females and males.

Contrary to the relationship between MHO and MHNW phenotypes discussed above, the MUO groups, regardless of gender, exhibited significantly higher age‐adjusted odds of elevated ASCVD risk compared to the MHNW phenotype. These observations support the synergistic impact of metabolic unhealthiness and obesity in contributing to cardiovascular risk, aligning with the concept that the combination of these factors may exert a more profound influence on cardiovascular outcomes than either factor [59, 60]. Furthermore, when obesity was defined by VAI, the MUHV phenotype exhibited even higher age‐adjusted odds of elevated ASCVD risk, signifying the independent contribution of visceral adiposity to cardiovascular risk and emphasising the need to incorporate novel markers such as VAI in risk assessment.

Though this study holds merit in its large sample size, comprehensive phenotyping, gender‐specific analysis and for being the first in studying the reported intricate associations in this ethnic population, the authors acknowledge some limitations worthy of mention. The cross‐sectional design of the study limits its applicability to establish causality. Long‐term longitudinal studies would be needed to establish the observations found in this study and will provide more robust insights into the temporal relationships between metabolic status, obesity phenotypes, and ASCVD risk. The study did not capture data on participants' physical activity levels, dietary habits or genetic factors, which may influence both metabolic status and obesity, and hence incorporating these lifestyle and genetic variables would enhance our understanding of individual susceptibility to ASCVD risks. Lastly, the study did not extensively explore underlying mechanistic pathways of the associations between phenotypes and ASCVD risk, and investigations into molecular and cellular mechanisms may further help in our understanding of how metabolic status and obesity interact to influence cardiovascular outcomes.

In conclusion, the observational study provides valuable insights into the interactions between metabolic state and obesity phenotypes, and the associated estimated 10‐year ASCVD risk scores in Saudi adults aged 40 to 79 years. We propose VAI as a better obesity index than BMI for identifying MHO phenotype. The results could help understand the discrepancy reported in the literature for this phenotype, referred to as the ‘MHO paradox’. The study also stresses the need to take individual metabolic phenotypes into account when assessing cardiovascular risk.

## Author Contributions

Conceptualization: K.W. Methodology: K.W. and S.S. Sample analysis: K.W. Data curation: K.W. Writing – original draft preparation: K.W. Writing – review and editing: S.S., B.K. and N.M.A.‐D. Supervision: S.S., B.K. and N.M.A.‐D. Project administration: K.W. Funding acquisition: N.M.A.‐D. All authors have read and agreed to the published version of the manuscript.

## Ethics Statement

The study was conducted according to the guidelines of the Declaration of Helsinki and approved by the Ethics Committee of the College of Medicine (E‐22‐7142, October 10, 2022), Ongoing Research Funding Program (ORF‐2025‐21), King Saud University, Riyadh, Saudi Arabia.

## Consent

Written informed consent was obtained from all participants involved in the study.

## Conflicts of Interest

The authors declare no conflicts of interest.

## Supporting information


**Table S1.** Bivariate correlation of ASCVD risk score with other parameters.
**Table S2.** ASCVD risk in phenotypes with respect to BMI + VAI.

## Data Availability

The data that support the findings of this study are available from the corresponding author upon reasonable request.
