# Supplementary material for: Exploring Metabolic Obesity Phenotypes and Atherosclerotic Cardiovascular Disease Risk in Arab Adults
Source: Clin Obes. 2025 Jun 27;15(6):e70032. doi: 10.1111/cob.70032 (PMC12603341; doi:10.1111/cob.70032)
Supplement: Supplementary file 1 — Table S1. Bivariate correlation of ASCVD risk score with other parameters. Table S2. ASCVD risk in phenotypes with respect to BMI + VAI. [file COB-15-e70032-s001.pdf]

Supplementary Table S1: Bivariate correlation of ASCVD risk score with other parameters

	**All**		**Females**		**Males**	
**Parameters**	**r**	**p**	**r**	**p**	**r**	**p**
Age	0.69	<0.001	0.77	<0.001	0.74	<0.001
BMI	0.08	<0.001	0.08	<0.001	0.09	<0.001
VAI	0.36	<0.001	0.39	<0.001	0.32	<0.001
Waist	0.17	<0.001	0.12	<0.001	0.15	<0.001
Hips	0.03	0.01	0.12	<0.001	0.07	0.00
WHR	0.12	<0.001	-0.01	0.60	0.12	<0.001
Systolic BP	0.42	<0.001	0.47	<0.001	0.38	<0.001
Diastolic BP	0.27	<0.001	0.28	<0.001	0.22	<0.001
Triglycerides	0.31	<0.001	0.25	<0.001	0.21	<0.001
Cholesterol	0.12	<0.001	0.13	<0.001	0.16	<0.001
HDL-Cholesterol	-0.42	<0.001	-0.33	<0.001	-0.31	<0.001
LDL-Cholesterol	0.15	<0.001	0.17	<0.001	0.15	<0.001
Glucose	0.37	<0.001	0.38	<0.001	0.36	<0.001
Insulin	0.11	<0.001	0.12	<0.001	0.07	0.06
HOMA-IR	0.25	<0.001	0.26	<0.001	0.20	<0.001

Note: The correlation coefficient was presented as "r" and its associated p-value was presented. P<0.05 was considered significant.

Table S2: ASCVD risk in phenotypes with respect to BMI + VAI

BMI + VAI	**All (5460)**	**Females (3519)**	**Males (1941)**	*p*	**10-year ASCVD risk (females)**				**10-year ASCVD risk (males)**			
					Low risk	Intermediate to High Risk	OR[a] **(95% CI)**	p	**Low risk**	Intermediate to High Risk	**OR[a] (95% CI)**	*p*
BMI (-), VAI (-)	254 (4.7)	126 (3.6)	128 (6.6)	<0.001	118 (93.7)	8 (6.3)			66 (51.6)	62 (48.4)		
BMI (+), VAI (-)	494 (9.0)	366 (10.4)	128 (6.6)		325 (88.8)	41 (11.2)	1.09 (0.7, 1.7)	0.719	58 (45.3)	70 (54.7)	0.54 (0.3, 0.9)	0.02
BMI (-), VAI (+)	566 (10.4)	300 (8.5)	266 (13.7)		240 (80.0)	60 (20.0)	1.63 (1.0, 2.6)	0.043	87 (32.7)	179 (67.3)	1.43 (0.9, 2.2)	0.1
BMI (+), VAI (+)	1997 (36.6)	1503 (42.7)	494 (25.6)		1141 (75.9)	362 (24.1)	1.15 (0.8, 1.8)	0.516	101 (20.4)	393 (79.6)	0.85 (0.6, 1.3)	0.41

Note: "+" and "–" refers to high VAI or obese and normal VAI or normal weight respectively.
